# Impact of overlapping fungal infection on the occurrence and prognosis of carbapenem-resistant gram-negative bacilli infection

**DOI:** 10.3389/fcimb.2025.1523233

**Published:** 2025-05-30

**Authors:** Jiahuan Li, Yushan Liu, Tingting Xu, Hongling Ma, Qian Zhang, Lijuan Xiong

**Affiliations:** ^1^ Department of Infectious Disease, Union Hospital, Tongji Medical College, Huazhong University of Science and Technology, Wuhan, Hubei, China; ^2^ Department of Clinical Laboratory, Union Hospital, Tongji Medical College, Huazhong University of Science and Technology, Wuhan, Hubei, China; ^3^ Department of Nosocomial Infection Management, Union Hospital, Tongji Medical College, Huazhong University of Science and Technology, Wuhan, Hubei, China

**Keywords:** carbapenem-resistant gram-negative bacilli, fungal infection, overlapping infection, risk factor, prognosis

## Abstract

**Background:**

The global threat of carbapenem-resistant gram-negative bacteria (CRGNB) infection is compounded by concurrent fungal infections, which present additional clinical challenges. This study aims to elucidate the impact of fungal infection on the occurrence and prognosis of CRGNB infection.

**Methods:**

We conducted a retrospective, single-center, observational cohort study of 2,273 patients with CRGNB and/or fungal infection from January 2018 to April 2023. Binary logistic regression analysis and multivariable Cox proportional hazards regression model were used to determine risk factors for the occurrence and prognosis of patients with CRGNB or fungal infections. Survival analysis was performed to investigate the impact of fungal co-infection on mortality of total GRGNB and bacterial subclasses infections.

**Results:**

Fungal infection was a independent risk factor for CRGNB infection (OR=1.381, *p*=0.015), and similarly, GRGNB was associated with an increased risk of fungal infection (OR=1.542, *p*<0.001). Besides, males and individuals with a history of ICU admissions, invasive surgeries, malignancies, mechanical ventilation, drainage tubes, or exposure to cephalosporin/carbapenem antibiotics were found to be more susceptible to both types of infections. Compared to patients with only GRGNB infection, co-infections contributed to a higher risk of mortality. However, co-infections do not amplify mortality risk in patients with only fungal infection. Further analysis revealed a significant increase in mortality of patients with carbapenem-resistant *Klebsiella pneumoniae* or carbapenem-resistant *Acinetobacter baumannii* co-infections, but no change in mortality rates was observed with carbapenem-resistant *Escherichia coli* or carbapenem-resistant *Pseudomonas aeruginosa*. Interestingly, we found that fungi were detected significantly earlier than CRGNB (median: 9 days vs. 21 days, *p*<0.001).

**Conclusions:**

In the current study, it was discovered that fungal infections preceded GRGNB infections and might contribute to the development of antibiotic resistance in some gram-negative bacteria, which ultimately leads to more severe clinical outcomes.

## 1 Introduction

Carbapenem-resistant gram-negative bacilli (CRGNB) poses a significant public health threat due to their widespread distribution and difficulty in containment (Zeng et al., 2023). Among the clinically important CRGNB, carbapenem-resistant *Klebsiella pneumoniae* (CRKP), carbapenem-resistant *Escherichia coli* (CREC), carbapenem-resistant *Acinetobacter baumannii* (CRAB), and carbapenem-resistant *Pseudomonas aeruginosa* (CRPA) are particularly concerning as they are classified as “urgent threat” pathogens by World Health Organization (WHO) and Centers for Disease Control and Prevention (CDC) (Tacconelli et al., 2018). These bacteria can cause severe infections, including bloodstream infections, severe pneumonia, complicated intra-abdominal infections, and urinary tract infections with poor prognosis and high morbidity/mortality rates (Pogue et al., 2022; Falcone et al., 2023; Gutiérrez-Gutiérrez et al., 2017).

The principal mechanisms involved in CRGNB resistance to carbapenems encompass enzyme production, deletion of outer membrane pore proteins, overexpression of efflux pumps, and biofilm formation (Suay-García and Pérez-Gracia, 2019; Munita and Arias, 2016; Azizi et al., 2015). In addition to the inherent characteristics of the organisms themselves, various clinical factors, such as intensive care unit (ICU) admission, invasive procedures, severe underlying diseases, history of hematological malignancies, multiple corticosteroid and immunosuppressant use, and prolonged/repeated broad-spectrum antibiotic administration, particularly cephalosporins and carbapenems, can influence the development and spread of CRGNB infections (Routsi et al., 2013; Gao et al., 2022).

Additionally, fungal infections can also affect the occurrence and treatment of CRGNB infections. Some patients with sepsis, chronic obstructive pulmonary disease, or severe immunodeficiency experience concurrent fungal and bacterial infections (Rolston et al., 2007; Cabrera-Rubio et al., 2012). *Candida albicans* is responsible for numerous nosocomial infections with a mortality rate exceeding 40% (Pfaller and Diekema, 2007). It forms biofilms on medical devices, which are resistant to the immune system and antibiotics (Ramage et al., 2002; Andes et al., 2004). The coexistence of *Candida albicans* and bacteria in mixed biofilms creates a complex microenvironment that impacts bacterial resistance. For instance, the presence of both *Candida albicans* and *Klebsiella pneumoniae* enhances the pathogenicity and proliferation of *Klebsiella pneumoniae* (Galdiero et al., 2021, 2020). *Aspergillus fumigatus*, another common conditional pathogenic fungus, causes over 200k cases of invasive aspergillosis annually and contributes a mortality rate above 50% (Brown et al., 2012). Cefepime alone or in combination with posaconazole exhibits reduced efficacy against mixed biofilms composed of *Pseudomonas aeruginosa* and *Aspergillus fumigatus* compared to *Pseudomonas aeruginosa* alone (Manavathu et al., 2014). Thus, concurrent fungal infections may exacerbate challenges associated with CRGNB infections. However, the risk and impact of overlapping fungal infection in CRGNB patients remain unclear.

By conducting this study, our objective is to identify risk factors associated with both the occurrence and mortality of CRGNB and/or fungal infections, clarify the correlation between fungal infection and the occurrence of CRGNB, and investigate the impact of these infections on patient prognosis.

## 2 Methods

### 2.1 Study design

This is a retrospective, single-center, observational cohort study. We reviewed medical records of patients admitted to the Huazhong University of Science and Technology Affiliated Union Hospital from January 2018 to April 2023. The study was approved by the ethics committee (No.2023-0792). Since this study is retrospective, the ethics committee decided that patient consent was not required.

### 2.2 Participants

**Figure 1:**
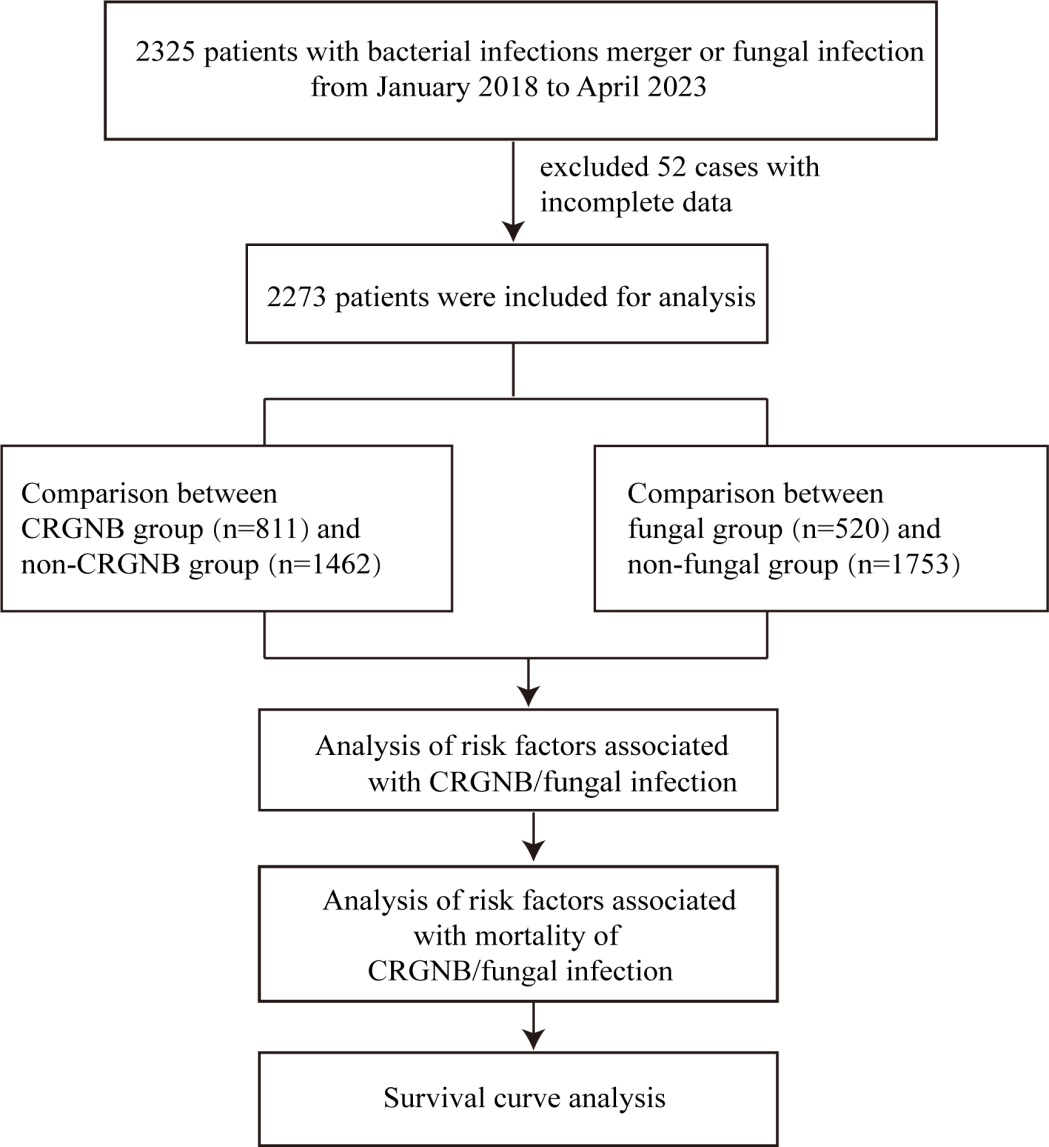
Flowchart of the study. CRGNB, carbapenem-resistant gram-negative bacilli.

The inclusion criteria for this study were: (a) patients over 18 years of age; and (b) patients tested positive for microbial culture after hospital admission, including: non-carbapenem resistant gram-negative bacteria (GNB), CRGNB, fungi infection, or GNB combined with fungi infection, regardless of whether GNB were CRGNB. CRGNB was defined as a gram-negative rod isolate that showed resistance to at least one carbapenem (including imipenem, meropenem, or ertapenem) with a minimum inhibitory concentration (MIC)≥4 µg/mL. Fungal infections are defined as those with host risk factors, clinical signs of infection and a positive microbiological definitive test result. Infections with strains other than GNB and fungi, and cases with missing key data were excluded from the study. According to whether the GNB is resistant, patients were divided into GRGNB and non-CRGNB groups. Depending on whether or not the infection is fungal, patients can be divided into fungal and non-fungal groups. Then, the risk factors and death risk factors were compared and survival curves were drawn for infected persons (**Figure 1**).

### 2.3 Data collection and exposures

After the exclusion of 52 non-compliant cases, 2273 patients were recorded in our study. The data recorded for 2273 patients included their age, gender, admission and discharge dates. Medical history data comprised underlying comorbidities such as diabetes, chronic obstructive pulmonary disease (COPD), pulmonary fibrosis, hypertension, coronary heart disease, heart failure, liver and kidney function failure, HIV infection, liver cirrhosis, rheumatic diseases, organ transplantation, neurological disorders, and tumors. Other medical history data included previous hospitalization history before detection of carbapenem-resistant bacteria, invasive surgeries, presence of indwelling invasive devices such as central venous catheters, urinary catheters or drainage tubes, mechanical ventilation, hemodialysis, ICU admission and length of stay, long-term use of steroids (>15 mg of prednisone daily for >2 weeks) or immunosuppressive drugs.

Within 24 hours of admission, patients were tested for white blood cell count, neutrophil count, procalcitonin (PCT) level, C-reactive protein (CRP) level, liver and kidney function indicators, cytokines, and other blood indicators. Imaging examinations, including cardiac ultrasound, head-lung-abdomen CT, etc., were performed to assist in identifying the site of infection.

Microbiological data were collected, including the species, time and source (such as blood, sputum, urine, drain fluid, pharyngeal swabs, stool, cerebrospinal fluid, wound secretions) of the first infection with GNB, fungi, and CRGNB. Antibiotic susceptibility test reports and culture time were recorded. Exposures to antibiotics (cephalosporins, carbapenems antibiotics) and antifungal drugs within 3 months were also collected. The main prognostic indicators such as length of hospital stay, outcome (improvement, aggravation, or death), 7-day and 28-day mortality were also collected. All data for this study were collected through an electronic medical record system. Antibiotic exposure was defined as receiving antibiotic treatment before microbial culture became positive.

Species identification was performed using the VITEK-2 system (Biomerieux, France), a fully automated microbial identification and susceptibility analysis system.The agar dilution method was applied to determine carbapenem susceptibility, whose results were interpreted following Clinical and Laboratory Standards Institute (CLSI) standards (Humphries et al., 2021). Carbapenem resistance was defined as resistance to meropenem or imipenem with a MIC≥4 µg/mL. Mycological criteria were positive direct microscopy with hyphae presented in specimen and positive culture. Direct microscopic examination was performed using a 20% KOH solution. Matrix-assisted laser desorption/ionization time-of-flight mass spectrometry (MALDI-TOF MS) is utilized for the rapid and accurate identification of fungal species. There were no changes in the microbiology laboratory techniques during the study period.

### 2.4 Outcome indicators

The primary outcome measures were risk factors and prognosis of CRGNB and fungal infection. The secondary outcomes were mortality risk of bacterial subclasses with fungal infections, and the time relationship between fungal infection and bacterial infection.

### 2.5 Statistical analysis

Categorical variables were described as counts and percentages, and compared using Pearson’s chi-square test or Fisher’s exact test. Continuous data was reported as mean±standard deviation or median with interquartile range (IQR), and group comparisons were performed using the Wilcoxon-Mann-Whitney test.

For CRGNB infection or fungal infection, all factors with *p*<0.05 were further analyzed using multivariable binary logistic regression analysis to determine independent risk factors. Odds ratios (ORs) with their 95% confidence intervals (CIs) and corresponding *p*-values were presented using a forest plot. Patients were followed up for 180 days by telephone, and factors with a significance level (*p*-value) less than 0.05 were further analyzed using a multivariable Cox proportional hazards regression model to investigate independent risk factors associated with mortality in patients infected with CRGNB or fungal pathogens.

Kaplan-Meier curves were used for survival analysis of patients. A Mann-Whitney U test was performed to evaluate any temporal relationship between the occurrence of fungal and bacterial infections. All statistical analyses were conducted using SPSS software (IBM Corp., Armonk, NY, USA, version 23.0), and a *p*-value<0.05 was considered statistically significant.

## 3 Results

### 3.1 Clinical and demographic characteristics of included patients

This study analyzed a total of 2,273 patients with the primary characteristics outlined in **Table 1**. Among them, there were 811 cases of CRGNB infections, 520 fungal infections, and 295 instances of co-infection with both CRGNB and fungi. The median age was 58 years (IQR: 49-68), and males accounted for 53.5% of the sample population. Prior to infection, approximately one-fifth (19.5%) had been admitted to the ICU and presented with more underlying conditions; within three months prior to admission, roughly one-quarter (24.5%) received cephalosporin antibiotics while another thirteen percent (13%) received carbapenem antibiotics. Most patients underwent invasive surgery or used indwelling catheters - some even using multiple catheters simultaneously - as part of their treatment regimen. The most common specimens collected included respiratory tract samples (n=1060), urine samples (n=798), blood samples (n=239), and secretion samples (n=137). Respiratory tract sources were identified as being responsible for most CRGNB infections (n=635), followed by secretions (n=73), blood (n=60) and urine (n=43). Similarly, respiratory tract sources also accounted for most fungal infections (n=473) followed by urine (n=36), blood (n=9) and secretion (n=3).

**Table 1:** The clinical characteristics of the included patients.

Characteristics	Total (N=2273)
**Male, n (%)**	1216 (53.5)
**Age, years, median (IQR)**	58.00 (49.00, 68.00)
**ICU admission, n (%)**	443 (19.5)
Underlying conditions, n (%)	
Diabetes	305 (13.4)
Cardiac insufficiency/heart failure	81 (3.7)
Other cardiovascular disease	787 (34.6)
Respiratory failure	128 (5.6)
Renal insufficiency/renal failure	135 (5.9)
Liver disease	51 (2.2)
Malignancy	170 (7.5)
Immunocompromised status	765 (33.7)
Fever	331 (14.6)
Sepsis/septic shock	76 (3.3)
Disorders of consciousness	43 (1.9)
Acute pancreatitis	19 (0.8)
**Invasive procedures, n (%)**	**1370 (60.3)**
Antibiotic exposure (within 3 months), n (%)	
Cephalosporin	557 (24.5)
Meropenem/Imipenem	295 (13.0)
Indwelling catheters, n (%)	
Urinary catheter	1209 (53.2)
Central venous catheter	481 (21.2)
Mechanical ventilation	838 (36.9)
Nasogastric tube	280 (12.3)
Drainage tube	911 (40.1)
Haemodialysis	157 (6.9)
Parenteral nutrition	276 (12.3)
**CRGNB infection, n (%)**	811 (35.7)
**Fungal infection, n (%)**	520 (22.9)

IQR, interquartile range; ICU, intensive care unit; CRGNB, carbapenem-resistant gram-negative bacilli.

Among the CRGNB infections, more than half of them was caused by *Acinetobacter baumannii* (n=554, 68.23%), followed by *Klebsiella pneumoniae* (n=114, 14.04%), *Pseudomonas aeruginosa* (n=51, 6.28%), and *Escherichia coli* (n=41, 5.05%). In addition to the four most common CRGNBs, the rest were clinically rare Gram-negative bacteria, including *Enterobacter clocloae*, *Enterobacter aerogenes*, *Stenotrophomonas maltophilia* and others, with a total of 51 cases, accounting for 6.28%. Among 520 patients with fungal infections, 39.2% (n=204) were infected with *Candida albicans*, followed by *Candida tropicalis* (n=85), *Aspergillus fumigatus* (n=60), *Aspergillus flavus* (n=57), *Candida parapsilosis* (n=50), and *Candida glabrata* (n=37). In addition, a small number of patients were simultaneously infected with two types of fungi, such as *Candida albicans* combined with *Candida tropicalis* (n=1), non-smooth *Candida parapsilosis* combined with *Aspergillus flavus* (n=1), *Aspergillus fumigatus* combined with *Aspergillus flavus* (n=3).

### 3.2 Risk factors associated with CRGNB and fungal infection

To identify risk factors for CRGNB infection, we categorized patients into two groups: 35.7% (n=811) in the CRGNB group and 64.3% (n=1462) in the remaining group (**Table 2**). Univariate analysis indicated that males and patients with a history of ICU admission were more susceptible to CRNGB. Multiple underlying diseases, such as tumors, organ failure, and sepsis, increased the risk of CRGNB infection. Invasive surgeries or recent exposure to cephalosporin and carbapenem antibiotics within three months also heightened the likelihood of CRGNB infection. Additionally, indwelling catheters other than urinary catheters and enteral hypernutrition increased the risk of CRGBN infection. Notably, fungal co-infection incidence was significantly higher in CRGNB-infected patients (36.4% vs 15.4%, *p*<0.001). Laboratory indicators showed that CRGNB-infected patients had elevated white blood cell counts, neutrophil counts, as well as higher levels of CRP and PCT. Furthermore, patients with CRGNB infection appeared to be more prone to blood hypercoagulation and albumin depletion.

**Table 2:** Univariate analysis of risk factors for CRGNB infection.

Characteristics	CRGNB (-) (n=1462)	CRGNB (+) (n=811)	*P-* value
**Male, n (%)**	637 (43.6)	579 (71.4)	<0.001
**Age, years, median (IQR)**	59.50 (50.00, 69.00)	58.00 (49.00,67.00)	0.021
**ICU admission, n (%)**	146 (10.0)	297 (36.6)	<0.001
Underlying conditions, n (%)			
Diabetes	212 (14.5)	93 (11.5)	0.042
Cardiac insufficiency/heart failure	36 (2.5)	45 (5.5)	<0.001
Other cardiovascular disease	506 (34.6)	281 (34.6)	0.985
Respiratory failure	38 (2.6)	90 (11.1)	<0.001
Renal insufficiency/renal failure	68 (4.6)	67 (8.3)	<0.001
Liver disease	38 (2.6)	13 (1.6)	0.124
Malignancy	133 (9.1)	37 (4.6)	<0.001
Immunocompromised status	413 (28.3)	352 (43.4)	<0.001
Fever	185 (12.7)	146 (18.0)	0.001
Sepsis/septic shock	20 (1.4)	56 (6.9)	<0.001
Disorders of consciousness	15 (1.0)	28 (3.4)	<0.001
Acute pancreatitis	7 (0.5)	12 (1.5)	0.012
**Invasive procedures, n (%)**	742 (50.8)	628 (77.4)	<0.001
Antibiotic exposure (within 3 months), n (%)			
Cephalosporin	273 (18.7)	284 (35.0)	<0.001
Meropenem/Imipenem	96 (6.6)	199 (24.5)	<0.001
Indwelling catheters, n (%)			
Urinary catheter	787 (53.8)	422 (52.0)	0.411
Central venous catheter	168 (11.5)	313 (38.6)	<0.001
Mechanical ventilation	294 (20.1)	544 (67.1)	<0.001
Nasogastric tube	90 (6.2)	190 (23.4)	<0.001
Drainage tube	445 (30.4)	466 (57.4)	<0.001
Haemodialysis	43 (2.9)	114 (14.0)	<0.001
Parenteral nutrition	172 (11.8)	104 (12.8)	0.459
**Fungal infection, n (%)**	225 (15.4)	295 (36.4)	<0.001

IQR, interquartile range; ICU, intensive care unit; CRGNB, carbapenem-resistant gram-negative bacilli.

Based on the presence of fungal infection, all patients were categorized into a fungal-infected group (n=520) and a non-fungal group (n=1753). The comparison between two groups is summarized in **Table 3**. Fungal-infected patients had a higher proportion of underlying diseases, such as cardiovascular disease, respiratory system disease, liver disease, malignant tumors, and immunodeficiency. They were more likely to acquire fungal infections after entering the ICU or undergoing invasive procedures or exposure to cephalosporin and carbapenem antibiotics within the previous three months. All indwelling catheters except for urinary catheters significantly increased the variability of fungal infections. Additionally, fungal-infected patients had a higher likelihood of co-existing CRGNB infection (56.7% vs 29.4%, *p*<0.001), as well as elevated white blood cell counts and neutrophil ratios with higher CRP and PCT levels.

**Table 3:** Univariate analysis of risk factors for fungal infection.

Characteristics	Fungi (-) (N=1753)	Fungi (+) (N=520)	*P-* value
**Male, n (%)**	844 (48.1)	372 (71.5)	<0.001
**Age, years, median (IQR)**	58.00 (48.00, 67.00)	63.00 (52.00, 72.00)	<0.001
**ICU admission, n (%)**	230 (13.1)	213 (41.0)	<0.001
Underlying conditions, n (%)			
Diabetes	229 (13.1)	76 (14.6)	0.362
Cardiac insufficiency/heart failure	40 (2.3)	41 (7.9)	<0.001
Other cardiovascular disease	568 (32.4)	219 (42.1)	<0.001
Respiratory failure	64 (3.6)	64 (12.3)	<0.001
Renal insufficiency/renal failure	79 (4.5)	56 (10.8)	<0.001
Liver disease	33 (1.9)	18 (3.5)	0.033
Malignancy	115 (6.6)	55 (10.6)	0.002
Immunocompromised status	556 (31.7)	209 (40.1)	<0.001
Fever	192 (11.0)	139 (26.7)	<0.001
Sepsis/septic shock	51 (2.9)	25 (4.8)	0.034
Disorders of consciousness	8 (0.4)	35 (6.7)	<0.001
Acute pancreatitis	12 (0.7)	7 (1.3)	0.146
**Invasive procedures, n (%)**	1035 (59.0)	335 (64.4)	0.028
Antibiotic exposure (within 3 months), n (%)			
Cephalosporin	317 (18.1)	240 (46.2)	<0.001
Meropenem/Imipenem	138 (7.9)	157 (30.2)	<0.001
Indwelling catheters, n (%)			
Urinary catheter	917 (52.3)	292 (56.1)	0.123
Central venous catheter	252 (14.4)	229 (44.0)	<0.001
Mechanical ventilation	510 (29.1)	328 (63.1)	<0.001
Nasogastric tube	131 (7.5)	149 (28.7)	<0.001
Drainage tube	656 (37.4)	255 (49.0)	<0.001
Haemodialysis	73 (4.1)	84 (16.15)	<0.001
Parenteral nutrition	186 (10.6)	90 (17.3)	<0.001
**CRGNB infection, n (%)**	516 (29.4)	295 (56.7)	<0.001

IQR, interquartile range; ICU, intensive care unit; CRGNB, carbapenem-resistant gram-negative bacilli.

**Figure 2:**
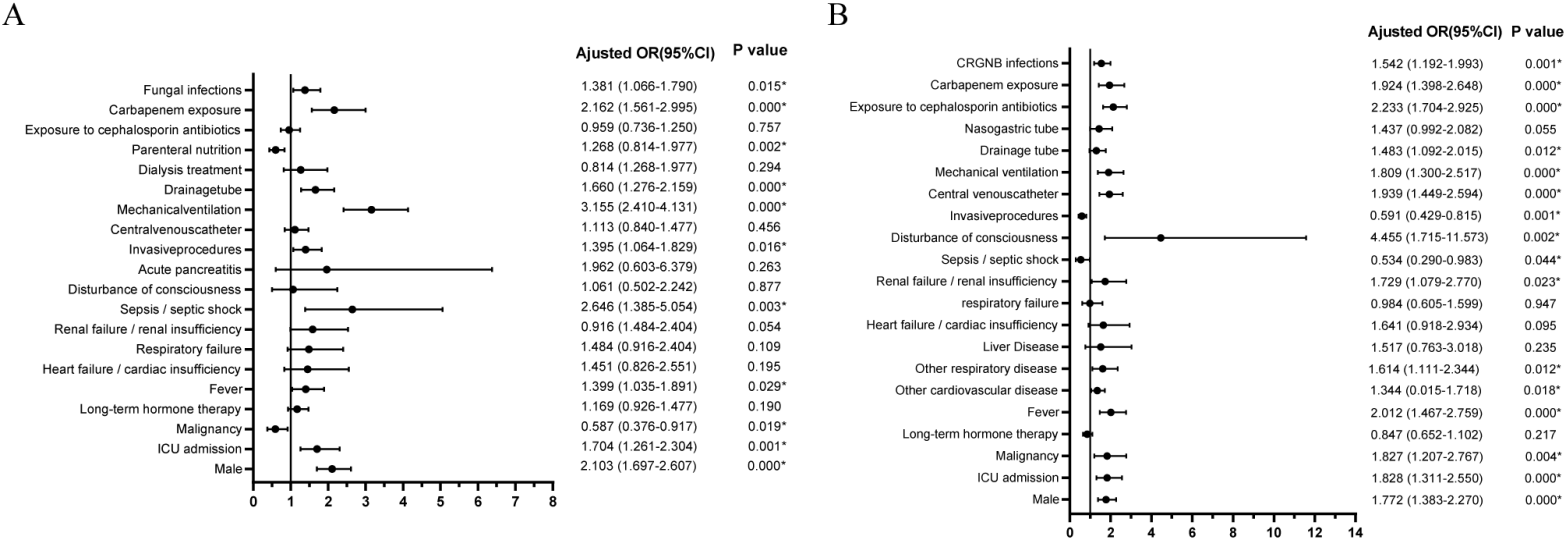
Multivariate logistic regression analysis of risk factors for CRGNB infection **(A)** or fungal infection **(B)**. CRGNB, carbapenem-resistant gram-negative bacilli; ICU, intensive care unit; OR, odds ratio.

When subjected to multivariate logistic analysis, it is noteworthy that co-infection with fungi independently increased the risk of CRGNB infection (odds ratio [OR]=1.381, 95% CI 1.066-1.790, *p*=0.015, **Figure 2A**), while being infected with CRGNB also independently increased the risk of fungal infection (OR=1.542, 95% CI 1.092-1.993, *p*=0.001, **Figure 2B**). Male gender, ICU admission, fever, invasive surgery, mechanical ventilation and exposure to carbapenem antibiotics were also identified as potential contributing factors for both infections (**Figures 2A, B**).

### 3.3 Mortality risk factors for CRGNB and fungal infection

The CRGNB-infected group was divided into a death group (n=134) and a survival group (n=677) for prognosis comparison. Univariate analysis revealed several factors associated with poor prognosis, including age, prior ICU admission, malignant tumors, fever, heart or renal failure, sepsis/septic shock, altered mental status, all invasive procedures, exposure to cephalosporin and carbapenem antibiotics in the previous three months, and fungal infection (**Table 4**).

**Table 4:** Univariate analysis of mortality risk factors for CRGNB infection.

Characteristics	Death group (n=134)	Survival group (n=677)	*P-* value
**Male, n (%)**	94 (70.1)	485 (71.6)	0.727
**Age, years, median (IQR)**	62.00 (54.00, 71.00)	57.00 (47.00, 66.00)	<0.001
**ICU admission, n (%)**	76 (56.7)	221 (32.6)	<0.001
Underlying conditions, n (%)			
Diabetes	21 (15.7)	72 (10.6)	0.095
Cardiac insufficiency/heart failure	19 (14.2)	26 (3.8)	<0.001
Other cardiovascular disease	59 (44.0)	222 (32.8)	0.012
Respiratory failure	20 (14.9)	70 (10.3)	0.123
Renal insufficiency/renal failure	26 (19.4)	41 (6.0)	<0.001
Liver disease	2 (1.5)	11 (1.6)	1
Malignancy	13 (9.7)	24 (3.5)	0.002
Immunocompromised status	56 (41.8)	296 (43.7)	0.68
Fever	46 (34.3)	100 (14.8)	<0.001
Sepsis/septic shock	19 (14.2)	37 (5.5)	<0.001
Disorders of consciousness	12 (9.0)	16 (2.4)	<0.001
Acute pancreatitis	3 (2.2)	9 (1.3)	0.685
**Invasive procedures, n (%)**	87 (65.0)	541 (80.1)	<0.001
Antibiotic exposure (within 3 months), n (%)			
Cephalosporin	72 (53.7)	212 (31.3)	<0.001
Meropenem/Imipenem	54 (40.3)	145 (21.4)	<0.001
Indwelling catheters, n (%)			
Urinary catheter	94 (70.1)	328 (48.4)	<0.001
Central venous catheter	80 (59.7)	233 (34.4)	<0.001
Mechanical ventilation	105 (78.4)	439 (64.8)	0.002
Nasogastric tube	59 (44.0)	131 (19.4)	<0.001
Drainage tube	59 (44.0)	407 (60.1)	0.001
Haemodialysis	36 (26.7)	78 (11.5)	<0.001
Parenteral nutrition	33 (24.6)	71 (10.5)	<0.001
**Fungal infection, n (%)**	69 (51.5)	226 (33.4)	<0.001

IQR, interquartile range; ICU, intensive care unit; CRGNB, carbapenem-resistant gram-negative bacill.

Similarly, the group infected with fungi was divided into a death group (n=118) and a survival group (n=402) to compare prognostic factors. In the univariate Cox regression analysis, risk factors associated with mortality of fungal infections are similar to those associated with CRGNB infection. However, concurrent CRGNB infection did not affect prognosis in patients with fungal infection (**Table 5**).

**Table 5:** Univariate analysis of mortality risk factors for fungal infection.

Characteristics	Death group (n=118)	Survival group (n=402)	*P-* value
**Male, n (%)**	78 (66.1)	294 (73.1)	0.137
**Age, years, median (IQR)**	65.00 (52.00, 74.00)	63.00 (52.00, 72.00)	0.006
**ICU admission, n (%)**	74 (62.7)	139 (34.6)	<0.001
Underlying conditions, n(%)			
Diabetes	18 (15.3)	58 (14.4)	0.823
Cardiac insufficiency/heart failure	23 (19.5)	18 (4.5)	<0.001
Other cardiovascular disease	61 (51.7)	158 (39.3)	0.017
Respiratory failure	28 (23.7)	36 (9.0)	<0.001
Renal insufficiency/renal failure	24 (20.3)	32 (8.0)	<0.001
Liver disease	3 (2.5)	15 (3.7)	0.738
Malignancy	22 (18.6)	33 (8.2)	0.001
Immunocompromised status	41 (34.7)	168 (41.8)	0.164
Fever	56 (47.4)	83 (20.6)	<0.001
Sepsis/septic shock	11 (9.3)	14 (3.5)	0.009
Disorders of consciousness	16 (13.6)	19 (4.7)	0.001
Acute pancreatitis	3 (2.5)	4 (1.0)	0.408
**Invasive procedures, n (%)**	61 (51.7)	274 (68.1)	0.001
Antibiotic exposure (within 3 months), n (%)			
Cephalosporin	73 (61.9)	167 (41.5)	<0.001
Meropenem/Imipenem	43 (36.4)	114 (28.3)	0.093
Indwelling catheters, n (%)			
Urinary catheter	86 (72.9)	206 (51.2)	<0.001
Central venous catheter	71 (60.1)	158 (39.3)	<0.001
Mechanical ventilation	89 (75.4)	239 (59.4)	0.002
Nasogastric tube	62 (52.5)	87 (21.6)	<0.001
Drainage tube	41 (34.7)	214 (53.2)	<0.001
Haemodialysis	33 (27.7)	51 (12.7)	<0.001
Parenteral nutrition	40 (33.9)	50 (12.4)	<0.001
**CRGNB infection, n (%)**	69 (58.5)	226 (56.2)	0.644

IQR, interquartile range; ICU, intensive care unit; CRGNB, carbapenem-resistant gram-negative bacilli.

**Figure 3:**
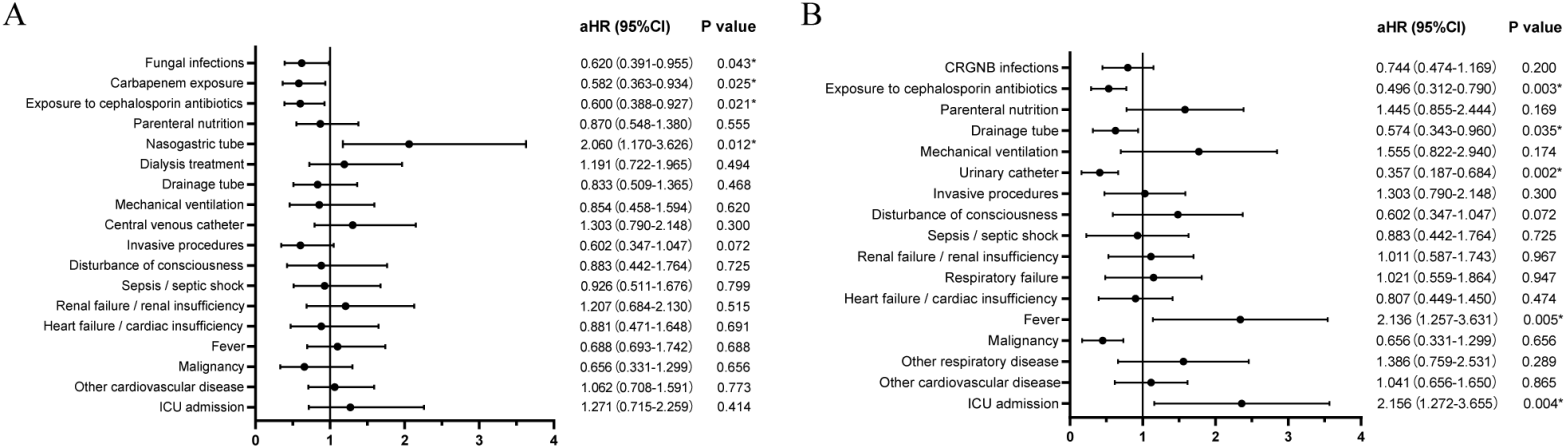
Risk factors for mortality of CRGNB infection **(A)** or fungal infection **(B)**. CRGNB, carbapenem-resistant gram-negative bacilli; ICU, intensive care unit; aHR, adjusted hazard ratio.

Concurrent fungal infection was found to be a significant risk factor for poor prognosis in multivariate Cox regression analysis (adjust hazard ratio [aHR]=0.620, 95% CI 0.391-0.955, *p*=0.043; **Figure 3A**). Nasogastric tube placement and exposure to cephalosporin antibiotics in the previous three months also impacted the prognosis of patients with CRGNB infection (**Figure 3A**). Fever, ICU admission, urinary catheter placement, and drainage tube placement influenced the prognosis of patients infected with fungi (**Figure 3B**).

### 3.4 Survival curve analysis

**Figure 4:**
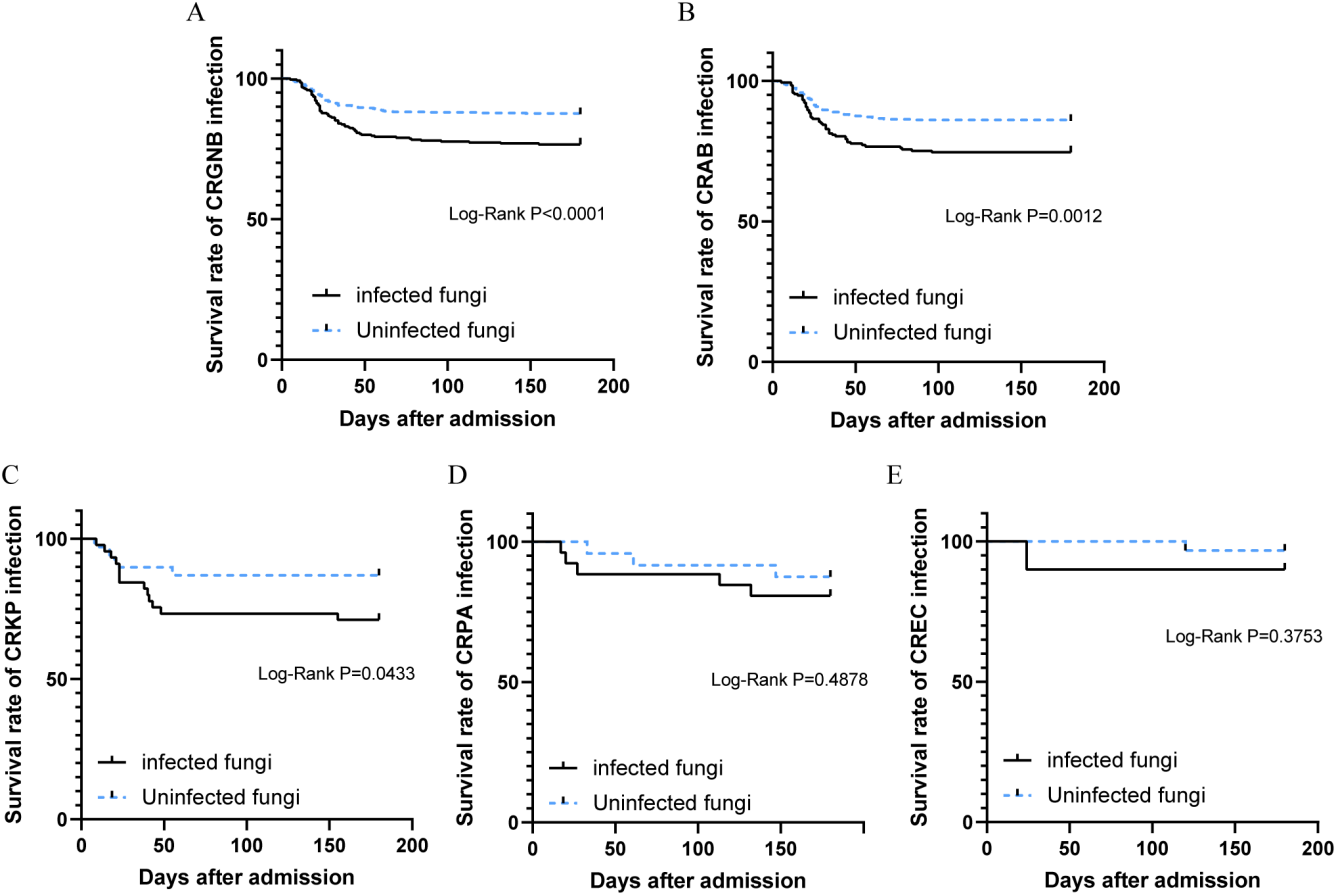
Survival analysis of patients with CRGNB overlapping fungal infections: **(A)** the overall GRGNB, **(B)** CRAB, **(C)** CRKP, **(D)**. CRPA, **(E)** CREC. CRGNB, carbapenem-resistant gram-negative bacilli; CRAB, carbapenem-resistant *Acinetobacter baumannii*; CRKP, carbapenem-resistant *Klebsiella pneumoniae*; CRPA, carbapenem-resistant *Pseudomonas aeruginosa*; CREC, carbapenem-resistant *Escherichia coli*.

Patients with concurrent CRGNB and fungal infections exhibited a significantly higher mortality rate compared to those without fungal infections (23.3% vs 12.6%, *p*<0.001, **Figure 4A**). Stratifying the survival curves by bacterial species revealed notable disparities in mortality rates for patients with overlapping fungal infections of CRAB or CRKP when compared to those without such co-infections (*p*=0.0012 and *p*=0.0433, **Figures 4B, C**). Nevertheless, there were no discernible discrepancies observed in the survival curves between patients concurrently infected with either CRPA or CREC and those lacking overlapping fungal infections (**Figures 4D, E**).

### 3.5 The time relationship between the occurrences of fungal infection and CRGNB infection

**Figure 5:**
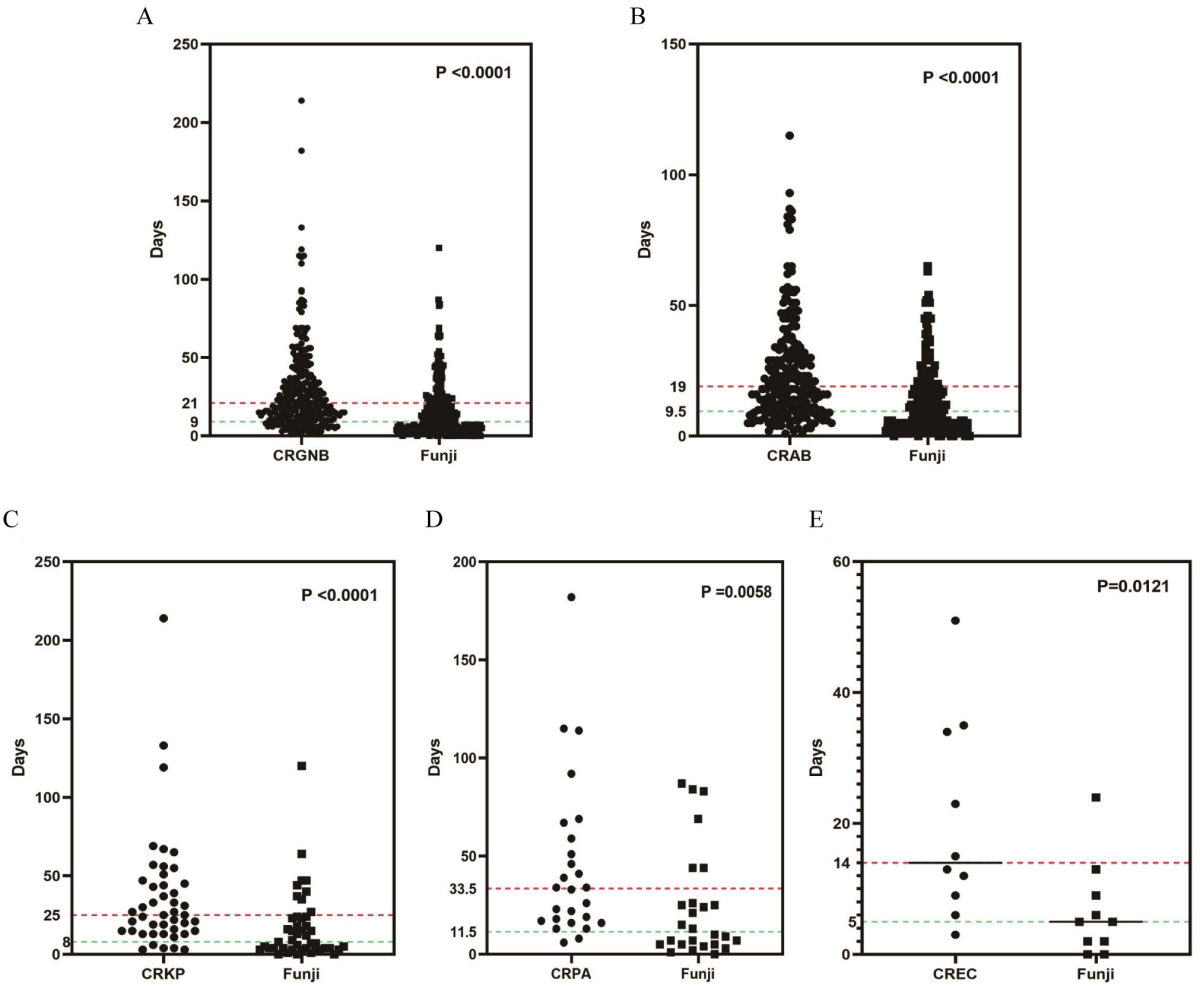
The time relationship between the occurrences of fungal infection and CRGNB infection: **(A)** the overall GRGNB, **(B)** CRAB, **(C)** CRKP, **(D)** CRPA, **(E)** CREC. CRGNB, carbapenem-resistant gram-negative bacilli; CRAB, carbapenem-resistant *Acinetobacter baumannii*; CRKP, carbapenem-resistant *Klebsiella pneumoniae*; CRPA, carbapenem-resistant *Pseudomonas aeruginosa*; CREC, carbapenem-resistant *Escherichia coli*.

We examined the time from admission to pathogen detection in 295 patients with concurrent CRGNB and fungal infections. The median time for detecting CRGNB was 21 days (IQR: 11-37), while for fungi it was 9 days (IQR: 4-21). There was a significant difference between the two groups (*p*<0.001, **Figure 5A**), indicating earlier detection of fungal infections compared to CRGNB infections. For patients with CRAB, CRKP, CRPA or CREC coexisting with fungal infection, fungi were also detected before bacteria (**Figures 5B-E**).

## 4 Discussion

To our knowledge, this study is the largest retrospective analysis to investigate the relationship between CRGNB and fungal infections, as well as their impact on patient outcomes. Our findings indicate that fungal infection increases the risk of CRGNB infection (OR=1.381, *p*=0.015), while GRGNB is associated with a higher risk of fungal infection (OR=1.542, *p*<0.001). Males and individuals with a history of ICU admissions, invasive surgeries, malignancies, mechanical ventilation, drainage tubes or exposure to cephalosporin/carbapenem antibiotics are more susceptible to both types of infections. Co-infections contribute to increased mortality in patients with GRGNB compared to those with only GRGNB infection. However, co-infections do not amplify mortality risk in patients with only fungal infection. Further analysis revealed a significant increase in mortality for patients with CRKB or CRAB co-infections, but no changes in mortality rates were observed for CREC or CRPA infections. Interestingly, our study suggests that fungal infections often precede GRGNB infections and may contribute to the development of antibiotic resistance in GNB, leading to more severe clinical consequences.

Currently, the issue of drug-resistant bacteria led by CRGNB is increasingly prominent and poses a significant threat to human health (Pogue et al., 2022; Falcone et al., 2023). A meta-analysis involving 92 studies reported that risk factors associated with CRGNB infection were previous antibiotic use, previous carbapenem use, previous colonization, mechanical ventilation, previous ICU stay, dialysis, catheter, length of stay in hospital, comorbidities, acute physiology and chronic health evaluation II (APACHE II), and intubation (Palacios-Baena et al., 2021). Another meta-analysis involving 34 studies found the risk factors associated with the highest risk for invasive *Candida* infection were broad-spectrum antibiotics (OR=5.6; 95% CI: 3.6-8.8), blood transfusion (OR=4.9; 95% CI: 1.5-16.3), *Candida* colonization (OR=4.7; 95% CI: 1.6-14.3), central venous catheter (OR=4.7; 95% CI: 2.7-8.1), and total parenteral nutrition (OR=4.6; 95% CI: 3.3-6.3) (Thomas-Rüddel et al., 2022). And previous antibiotic use (OR=9.3; 95% CI: 3.2-27.0) and bacterial infections (OR=4.3; 95% CI: 2.1-8.6) is a risk factor for invasive candidiasis in liver transplant patients (Phoompoung et al., 2022). Our study has identified multiple risk factors associated with CRGNB and fungal infection, which are mostly consistent with these researches. Organ failure and tumors are always associated with higher bacterial and fungal infections and mortality, which are associated with organ dysfunction, drug metabolism disorders, decreased immunity and other factors (Mezzano et al., 2022; Van de Louw et al., 2019; Zhuang et al., 2022). For patients with malignancies, chemotherapy-induced neutropenia is one of the most common complications, which greatly debilitates the patient’s immunity and increases the chance of infection (El-Mahallawy et al., 2015). One study conducted in Iran has shown that the overall mortality rate of tumor patients with bacterial bloodstream infection is as high as 21.5% (Amanati et al., 2021). Invasive procedures, such as tracheal intubation and mechanical ventilation, can compromise the integrity of skin and mucosal barriers, facilitating pathogen entry into the body and increasing the risk of infection (Rello et al., 2014; Zheng et al., 2020). The use of cephalosporin and carbapenem antibiotics has been linked to the development of drug-resistant genes and promotion of CRGNB infections (Yang et al., 2022), disrupting the balance of intestinal flora. This disruption leads to impaired IL-17A and GM-CSF mediated antifungal immunity in the gut, resulting in bacterial translocation in intestines along with fungal co-infections (Drummond et al., 2022). ICU patients face an elevated risk of resistant gram-negative bacterial and fungal infections due to critical illness, compromised immunity, device utilization, and extensive antibiotic treatment.

Previous study found that the proportion of patients with bacteria-fungus mixed infection was as high as 40.3% among patients with pulmonary fungal infection, and co-infection may worsen the disease, which may indicate a positive correlation between interspecific microbial interactions (Zhao et al., 2021). In our multifactorial analysis, we found that fungal infections and CRGNB infections were independent risk factors; however, it is noteworthy that fungal infections often precede the occurrence of CRGNB infections suggesting fungi might contribute to bacterial drug resistance development.

Patients with CRGNB infections have a significantly worse prognosis and higher mortality risk compared to those infected by susceptible microorganisms, as evidenced by numerous prior studies (Gao et al., 2022; Ahn et al., 2023). However, limited research exists on the complexities of CRGNB when combined with fungal infections. Our findings indicate that the presence of fungal infection significantly increases the risk of death for patients. This may be attributed to fungi’s impact on bacterial growth and their response to antibiotics. Studies have shown that *Candida albicans*’ β-1,3-Glucan affects *Escherichia coli*’s susceptibility to certain antibiotics (De Brucker et al., 2015). Additionally, bacteria can form polymicrobial biofilms with fungi to protect themselves, consequently affecting antibiotic permeability and therapeutic efficacy (Lohse et al., 2018).

Biofilm is a microorganism community that forms on living or non-living surfaces with extracellular polymeric substance (EPS), which has a unique three-dimensional structure and function. EPS, a major component of biofilms, blocks drug diffusion in polymicrobial biofilms (Karygianni et al., 2020), allowing bacteria to upregulate their antimicrobial resistance genes. Also, environmental and metabolic changes in EPS affect the growth and gene expression of pathogenic bacteria in polymicrobial biofilms, resulting in more antimicrobial resistance genes (Galdiero et al., 2021; Harriott and Noverr, 2009). Both bacteria and fungi can form biofilms and often coexist in polymicrobial ones. *Candida albicans* is important for forming such structures. Medical devices like stents, catheters, tracheal tubes, and pacemakers favor biofilm development. When these polymer surfaces meet suitable conditions, *Candida albicans* can initiate highly structured biofilm formation (Lohse et al., 2018). Compared to single-species biofilms infections, polymicrobial ones are more invasive and resistant to conventional antimicrobial therapies (Galdiero et al., 2021; de Alteriis et al., 2018). This complexity poses challenges for diagnosis and treatment, leading to worse clinical outcomes.

Previous studies have shown synergistic effects between *Candida albicans* and *Klebsiella pneumoniae* (Galdiero et al., 2021, 2020). The formation of a biofilm by *Candida albicans* creates an oxygen-depleted microenvironment through glucose consumption, promoting the proliferation of facultative anaerobes and enhancing the virulence of *Klebsiella pneumoniae* (Galdiero et al., 2021; Fox et al., 2014). This may partially explain the increased mortality observed in patients co-infected with *Candida albicans* and CRKP. Similarly, *Candida albicans* biofilms can also contribute to drug resistance in *Escherichia coli*. A study by Eshima S suggests that meropenem tolerance in *Escherichia coli* is partly attributed to the formation of a dual biofilm with *Candida albicans in vitro* (Eshima et al., 2022). However, our study found no significant difference in mortality rates among patients with co-infections compared to those with simple CREC infections, possibly due to delayed onset of fungal infections.

On the other hand, not all bacteria-fungi interactions are synergistic. Multiple studies have confirmed the antagonistic interaction between *Candida albicans* and bacteria, such as *Pseudomonas aeruginos*a, *Proteus*, and *Salmonella typhimurium* during biofilm formation (Hogan and Kolter, 2002; Kim and Mylonakis, 2011; Kart et al., 2020). *Pseudomonas aeruginos*a has been reported to form a dense biofilm on *Candida albicans* filaments and kills the fungus (Hogan and Kolter, 2002). *Pseudomonas aeruginosa* lipopolysaccharide has also been shown to inhibit *Candida albicans* biofilm formation and hyphal development by targeting glycolysis-associated mechanisms during candidal filamentation (Bandara et al., 2013). Additionally, in mixed biofilms, *Pseudomonas aeruginosa* competes with *Candida albicans* for iron and reduces the metabolic activity of the fungus (Purschke et al., 2012). These findings partly explain why co-infection with *Pseudomonas aeruginosa* does not lead to increased mortality in fungal infections. However, it is worth mentioning that latest research found that *Candida albicans* sequestered essential magnesium ions from the *Pseudomonas aeruginosa* and Mg^2+^ limitation enhanced bacterial resistance to polymyxin antibiotics like colistin (Hsieh et al., 2024), suggesting that the fungi plays a part in drug resistance of the bacterial. The interaction of *Acinetobacter baumannii* with fungi is controversial. Some studies have demonstrated that *Acinetobacter baumannii* can adhere to the mycelium of *Candida albicans*, inhibiting its growth and reducing its virulence through the production of OmpA proteins (Gaddy et al., 2009; Peleg et al., 2008). However, it has also been observed that *Acinetobacter baumannii* can thrive in the presence of another yeast species called *Saccharomyces cerevisiae* (Smith et al., 2004). In rats infected with *Acinetobacter baumannii* and colonized by *Candida albicans* in their respiratory tract, higher bacterial counts and more severe pneumonia were observed (Tan et al., 2016). This study found that co-infection with fungi increased CRAB mortality rates, indicating a general contribution of fungi to its pathogenicity.

Our study had several limitations. Firstly, it was conducted using data from a single center, which may limit its generalizability to other institutions. However, the large sample size increased the confidence of our findings. Secondly, as a retrospective study, accurately distinguishing between infection and colonization was challenging. However, we used multiple indicators and had the results confirmed by senior infectious disease doctors. Thirdly, while our study did not assess the sensitivity and effectiveness of antimicrobial therapy, this should be a focus of our future research, given the close relationship between patient survival and proper treatment.

## 5 Conclusion

In our study, we found that fungal infections preceded GRGNB infections. And co-infections with fungi contributed to increased mortality in patients with GRGNB compared to those with only GRGNB infection, especially in patients with CRKB or CRAB co-infections. Our paper highlighted the impact of fungal infections on the occurrence of CRGNB and the prognostic differences in various co-infections. This underscores need to prevent fungal infections to reduce CRGNB occurrences. Given the high mortality rate and challenges in antibiotic treatment for CRGNB infections, our findings offer valuable insights for clinical management.

## Data Availability

The original contributions presented in the study are included in the article/supplementary material. Further inquiries can be directed to the corresponding authors.
